# β3 Adrenoceptor Agonism Prevents Hyperoxia-Induced Colonic Alterations

**DOI:** 10.3390/biom13121755

**Published:** 2023-12-06

**Authors:** Luca Filippi, Patrizia Nardini, Virginia Zizi, Marta Molino, Camilla Fazi, Maura Calvani, Francesco Carrozzo, Giacomo Cavallaro, Giorgia Giuseppetti, Laura Calosi, Olivia Crociani, Alessandro Pini

**Affiliations:** 1 Department of Clinical and Experimental Medicine, University of Pisa, 56124 Pisa, Italy; g.giuseppetti@studenti.unipi.it; 2 Department of Experimental and Clinical Medicine, University of Florence, 50139 Florence, Italy; patrizia.nardini@unifi.it (P.N.); virginia.zizi@unifi.it (V.Z.); marta.molino@unifi.it (M.M.); laura.calosi@unifi.it (L.C.); olivia.crociani@unifi.it (O.C.); 3 Imaging Platform, Department Experimental and Clinical Medicine, University of Florence, 50139 Florence, Italy; 4 Department of Pediatric, Meyer Children’s University Hospital, 50139 Florence, Italy; camilla.fazi@unifi.it; 5 Division of Pediatric Oncology/Hematology, Meyer University Children’s Hospital, 50139 Florence, Italy; maura.calvani@meyer.it (M.C.); francesco.carrozzo@unifi.it (F.C.); 6 Neonatal Intensive Care Unit, Fondazione IRCCS Ca’ Granda Ospedale Maggiore Policlinico, 20122 Milan, Italy; giacomo.cavallaro@policlinico.mi.it

**Keywords:** beta-3 adrenoceptor, beta-3 adrenoceptor agonism, BRL37344, hyperoxia, colon, gut, development, proximal colon

## Abstract

Oxygen level is a key regulator of organogenesis and its modification in postnatal life alters the maturation process of organs, including the intestine, which do not completely develop in utero. The β3-adrenoreceptor (β3-AR) is expressed in the colon and has an oxygen-dependent regulatory mechanism. This study shows the effects of the β3-AR agonist BRL37344 in a neonatal model of hyperoxia-driven colonic injury. For the first 14 days after birth, Sprague–Dawley rat pups were exposed to ambient oxygen levels (21%) or hyperoxia (85%) and treated daily with BRL37344 at 1, 3, 6 mg/kg or untreated. At the end of day 14, proximal colon samples were collected for analysis. Hyperoxia deeply influences the proximal colon development by reducing β3-AR-expressing cells (27%), colonic length (26%) and mucin production (47%), and altering the neuronal chemical coding in the myenteric plexus without changes in the neuron number. The administration of BRL37344 at 3 mg/kg, but not at 1 mg/kg, significantly prevented these alterations. Conversely, it was ineffective in preventing hyperoxia-induced body weight loss. BRL37344 at 6 mg/kg was toxic. These findings pave the way for β3-AR pharmacological targeting as a therapeutic option for diseases caused by hyperoxia-impaired development, typical prematurity disorders.

## 1. Introduction

The morphogenetic processes during mammalian ontogeny take place in the hypoxic environment of the uterus [1], where low oxygen tension (2–8%) is required to guide the mechanisms of cell proliferation and differentiation [2]. After birth, exposure to ambient oxygen levels (21%) promotes the final stage of the maturation process of many organs, including the lungs, eyes and gut, which do not completely develop in utero [3,4,5,6]. Therefore, oxygen level is a critical regulator of correct organogenesis both in prenatal and postnatal life, and its alteration could significantly derail the normal developmental sequence of organs, impairing their morpho-functional characteristics and promoting injury [7]. For instance, supplemental oxygen therapy, often used in clinical practice to treat neonatal respiratory disorders, sometimes produces too high an oxygen level (hyperoxia) that causes adverse effects, playing a pivotal role in the development of bronchopulmonary dysplasia (BPD), retinopathy of prematurity (ROP) [8] and gut alterations [5] that may lead to necrotizing enterocolitis (NEC). Regarding the latter organ, a growing body of evidence has indicated that hyperoxia exposure modifies the distal small intestine of newborn rats, altering the mucosal structure [5,8] and unbalancing the microflora [9]. In particular, it has been demonstrated that hyperoxia, by increasing the generation of reactive oxygen species (ROS), disrupts the intestinal barrier of newborn rats, compromising the integrity of the tight junctions that connect the surface epithelial cells [5,9]. However, to our knowledge, little is known about the effect of neonatal hyperoxia in the large intestine, particularly in the colon, except for a single report of wall-thickening [8].

Among the numerous signaling pathways that oxygen may activate during organogenesis, oxygen-sensing mechanisms triggered by catecholamines have been proposed to play a relevant role [10]. The effects of catecholamines are mediated by β-adrenoreceptors (β-ARs), which are G-protein-coupled receptors classified as α1-ARs, α2-ARs, and β1-Ars, β2-Ars and β3-ARs. Compared to the other β-AR subtypes, the β3-adrenoreceptor, first discovered in adipose tissue in 1989 [11], shows an oxygen-dependent regulatory mechanism [12], being up-regulated under hypoxia by hypoxia-inducible factor (HIF)-1 [13], the first characterized oxygen-dependent transcriptional activator [14]. In particular, in a mouse model of retinopathy, HIF-1 has recently been demonstrated to increase β-AR expression by directly binding to its enhancer region [14]. Moreover, β3-AR expression is high in the developing embryo during hypoxic intra-uterine life [15,16,17], while it decreases after delivery [18] when newborns are suddenly exposed to ambient oxygen levels. During adult life, β3-AR persists in restricted human adult districts [14,19], including the colon [20], where it is present on cholinergic neurons in both the submucosal and myenteric plexuses of the enteric nervous system (ENS), and is thought to be involved in visceral sensitivity [20]. On these grounds, considering the relationship between oxygen levels and β3-AR expression, it is conceivable that high oxygen levels may downregulate β3-AR expression, thus legitimating an exploration of the hypothesis of β3-AR’s involvement in neonatal hyperoxia-induced colonic alterations.

To substantiate this hypothesis, we evaluated the effects of BRL37344, a commonly used selective β3-AR agonist, in a neonatal rat model of hyperoxia-driven colonic injury. The reported findings, although new and promising, were obtained from an exploratory study and need to be further confirmed by a formal hypothesis-testing study.

## 2. Materials and Methods

### 2.1. Oxygen Exposure and Drug Administration

The study was approved by the Ethical Committee of Florence University and the Italian Ministry of Health (Authorization N. 453/2021). Time-dated pregnant Sprague–Dawley rats (Crl:CD(SD)) (RRID: RGD_734476) were purchased from Charles River Laboratories (Wilmington, MA, USA) and housed in individual cages under constant environmental and nutritional conditions at 25 ± 2 °C, with 12 h alternating light and dark cycles and ad libitum access to food and water. Immediately after delivery, litters were pooled and randomly distributed among the mothers within 4 h of birth. The pups were then assigned to either ambient oxygen levels (normoxia, 21%) or oxygen-enriched atmosphere (hyperoxia, 85%) treatments. To avoid oxygen toxicity in nursing mothers, they were rotated between oxygen treatment and normoxia litters every 24 h. Hyperoxia was maintained in a semi-sealable plexiglass animal chamber (BioSpherix, Parish, NY, USA) and oxygen level was monitored using a ProOx P110 Cytocentric O_2_ Controller (BioSpherix, Parish, NY, USA). The pups were divided into six groups: (1) normoxia (normoxia control group), where the animals were reared with 21% oxygen and untreated; (2) normoxia with BRL37344, where the pups were reared with 21% oxygen and treated with 3 mg/kg BRL37344 (Merck, Darmstadt, Germany); (3) hyperoxia (hyperoxia control group), where the animals were exposed to 85% oxygen and untreated; (4) hyperoxia with BRL37344 (1 mg/kg), where the pups were reared to 85% oxygen and treated with 1 mg/kg BRL37344; (5) hyperoxia with BRL37344 (3 mg/kg), where the neonatal rats were exposed to 85% oxygen and treated with 3 mg/kg BRL37344; (6) hyperoxia with BRL37344 (6 mg/kg), where the pups were reared to 85% oxygen and treated with 6 mg/kg BRL37344. The β3-AR agonist BRL37344 was administered subcutaneously from the day of birth (P0) to day 14 (P14) every 12 h. Body weights were measured daily. At the end of the experimental period, rats were sacrificed by the intraperitoneal injection of sodium pentobarbital (800 mg/kg) and proximal colon specimens were carefully excised, their length was measured, and they were washed in 0.1 M phosphate-buffered saline (PBS) pH 7.4 and collected for biochemical and morphologic evaluations. Treatment with BRL37344 at 6 mg/kg resulted in the death of all animals exposed to hyperoxia within the first week. To ensure a constant oxygen level for the other experimental groups, the hyperoxic chamber was only opened twice a day during administrations and, for this reason, no tissue sampling could be performed, and no data are available from the group of hyperoxia treated with BRL37344 at 6 mg/kg, except mortality data.

### 2.2. Flow Cytometric Analysis

Flow cytometry analysis was performed on the colon cells of at least 19 rats per group. The tissues were homogenized with the Multi Tissue Dissociation Kit 2 (#130-110-203, Miltenyi Biotec, Bergisch Gladbach, Cologne, Germany) and cells were isolated from the total cell suspension. Cells were then immunolabeled with the fluorochrome-conjugated antibodies anti-β3-AR A594 (bs-10921R, Bioss, Woburn, MA, USA. RRID: AB_2927635) and anti-CD45 [OX-1] FITC (GTX43587, GeneTex, Irvine, CA, USA. RRID: AB_11175386), diluted 1:50. The labeled cells were analyzed using a MACSQuant Analyzer 10 Flow Cytometer (Miltenyi Biotec, Bergisch Gladbach, Cologne, Germany), and data were processed using Flowlogic 8.7 software (Miltenyi Biotec, Bergisch Gladbach, Cologne, Germany). To validate the anti-β3-AR antibody, rat epidermal and fat samples were used as negative and positive controls, respectively (Figure S1, Supplementary Materials).

### 2.3. Histological Examinations

Proximal colonic specimens (2 cm from the cecum) were fixed overnight in 4% paraformaldehyde, embedded in paraffin, and cut with a microtome in 5 μm thick sections; these were stained with either hematoxylin and eosin (H/E) to analyze gut morphology or PAS reaction to evaluate the production of mucins. Examinations were carried out with a light Zeiss Axioskop microscope (Zeiss, Mannheim, Germany) equipped with 10× and 40× objectives by two independent observers (A.P., P.N.) blinded to the experimental groups. PAS-positive area was evaluated quantitatively with the ImageJ bundled with Zulu OpenJDK 13.0.6 software (NIH, Bethesda, ML, USA) by thresholding the positive tissue areas on standard regions of interest (ROI) and reported as the percentage of positive area/total area of ROI.

### 2.4. Immunofluorescence Analysis

The immunofluorescence analysis was performed on rehydrated sections rinsed in Tris buffer (10 mM) with EDTA (1 mM, pH 9.0) or in sodium citrate buffer (10 mM, pH 6) for 20 min at 90–92 °C for antigen retrieval. The sections were washed in PBS, blocked with 1.5% bovine serum albumin (BSA, Applichem, Darmstadt, Germany) in PBS to minimize non-specific binding and incubated at 4 °C overnight with the primary antibodies listed in Table 1. To validate the anti-β3-AR antibody, rat epidermal and fat samples were used as negative and positive controls, respectively (Figure S2, Supplementary Materials). For the double immunofluorescent stainings—protein gene product 9.5 (PGP9.5) and neuronal nitric oxide synthase (nNOS) or choline acetyltransferase (ChAT), and β3-AR and ChAT or glial fibrillary acidic protein (GFAP)—the sections were incubated overnight with a mixture of the 2 primary antibodies raised in different species, diluted in PBS with 1% BSA. The sections were then treated for 2 h at room temperature with appropriate fluorochrome-conjugated secondary antibodies diluted in BSA 0.15% in PBS. Subsequently, the specimens were rinsed with PBS and mounted in an anti-fade aqueous medium (FluoroshieldTM with DAPI, Thermo Fisher Scientific, Waltham, MA, USA). Primary antibody omission was used as the negative control. The immunolabeled sections were observed under a Leica Stellaris 5 confocal microscope (Leica Microsystem, Wetzlar, Germany), equipped with Leica Plan Apo 63X/1.43NA oil immersion objective (Leica Microsystem, Wetzlar, Germany).

The total number of PGP9.5-, nNOS-, and ChAT-immunoreactive cells was measured within the myenteric ganglia along the entire section by two independent observers (A.P., P.N.) blind to the experimental groups. The results are expressed as a percentage of nNOS- or ChAT- and PGP9.5-positive cells per section (mean ± S.D. of at least 3 sections/animal). The quantification of SP-positive area, assumed as a marker of nerve fibers, was morphometrically assessed within the myenteric ganglia on digitized images using the threshold tool of ImageJ bundled with Zulu OpenJDK 13.0.6 software (NIH, Bethesda, ML, USA) (at least 3 sections/animal). The results are expressed as the ratio between the SP-positive area and the total area of myenteric ganglia considered in the measurements.

### 2.5. Statistical Analysis

Data are expressed as mean ± S.D. Statistical analysis was performed by the non-parametric Kruskal–Wallis test. The normoxia + BRL37344 group was compared to the normoxia group (#), and the hyperoxia +BRL37344 groups were compared to both the hyperoxia (§) and normoxia groups (°), to evaluate the degree of protection exerted by BRL37344 from the adverse effects of high oxygen levels. Statistical analysis was performed using GraphPad Prism 9.0 software (GraphPad, San Diego, CA, USA).

## 3. Results

### 3.1. β3-AR Expression on Colonic Cells

**Figure 1:**
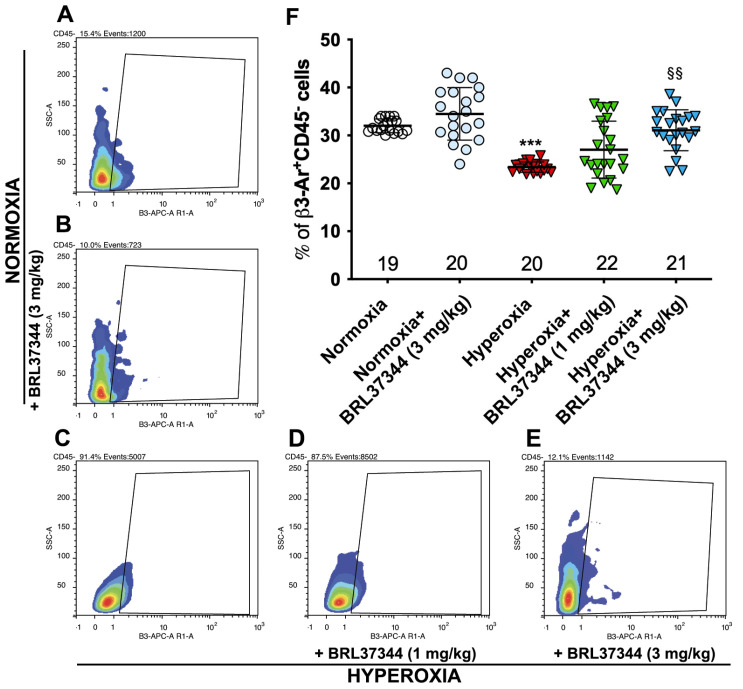
Effect of hyperoxia on β3-AR expressing cells. (**A**–**E**) Flow cytometric plot showing colonic cells expressing β3-AR (β3-AR^+^ CD45^−^ cells). (**F**) Analysis of the cytofluorimetric plots. Hyperoxia exposure significantly reduced the number of resident colonic cells expressing β3-AR, while treatment with BRL37344 at 3 mg/kg, but not at 1 mg/kg significantly prevented this alteration. No differences were found in the rats treated or not with the β3-AR agonist in normoxic conditions. Values are expressed as mean ± S.D. *** *p* < 0.001 hyperoxia versus normoxia; §§ *p* < 0.01 hyperoxia + BRL37344 (3 mg/kg) versus hyperoxia.

To investigate whether hyperoxia affects β3-AR expression via resident colonic cells, flow cytometric analysis was performed on suspensions of whole cells isolated from the colonic samples upon the exclusion of inflammatory cells, which constitutively express β3-AR [21,22], as identified by the expression of the pan-hematopoietic marker CD45 [23]. As shown in Figure 1, compared with pups reared to normoxia, a statistically significant decrease in the number of colonic cells expressing β3-AR (β3-AR^+^ CD45^−^cells) was found in pups from the hyperoxia control group (Figure 1, panels A, C, F). Treatment with BRL37344 at 3 mg/kg prevented a hyperoxia-induced reduction in the number of cells expressing β3-AR (Figure 1, panels A, C–F), whereas the lowest 1 mg/kg dose was ineffective. Of note, the highest BRL37344 (6 mg/kg) resulted in the death of all animals exposed to hyperoxia within the first week, thus preventing any measurements of considered markers. Treatment with the β3-AR agonist under normoxia did not affect the number of resident colonic cells expressing β3-AR (β3-AR^+^ CD45^−^ cells) (Figure 1, panels A, B and F).

### 3.2. Body Weight

As shown in Table 2, at P0 the body weight of the pups of all experimental groups was comparable, whereas at P14 the body weight of hyperoxia-exposed rats was significantly lower than that of normoxia-exposed rats; treatment with BRL37344 at both 1 and 3 mg/kg did not prevent weight loss. As expected, the administration of BRL37344 at 3 mg/kg to the normoxia-exposed pups did not affect their body weight.

### 3.3. Colon Length and Mucosal Morphology

**Figure 2:**
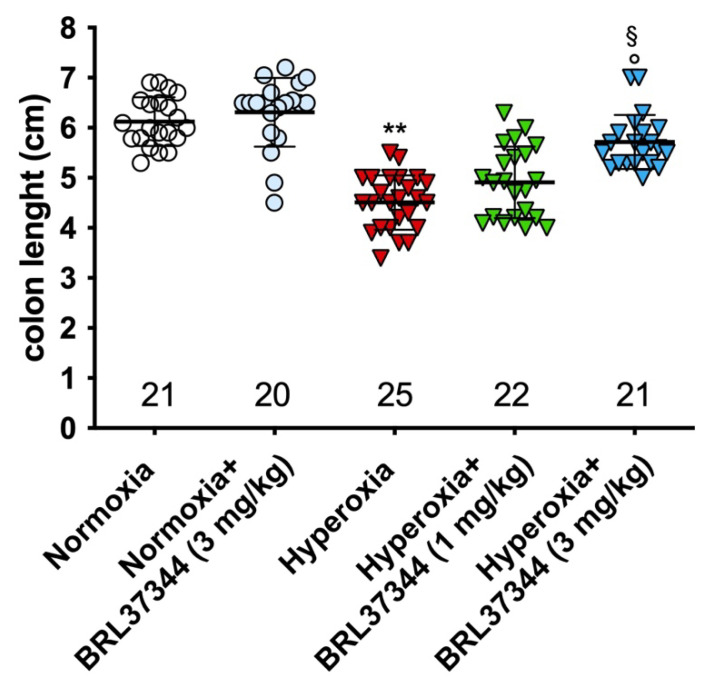
Effect of hyperoxia on colon length. Hyperoxia exposure significantly reduced the colon length of pups and treatment with BRL37344 at 3 mg/kg significantly prevented this alteration. Values are expressed as mean ± S.D. ** *p* < 0.01 hyperoxia versus normoxia; § *p* < 0.05 hyperoxia + BRL37344 (3 mg/kg) versus hyperoxia; ° *p* < 0.05 hyperoxia + BRL37344 (3 mg/kg) versus normoxia.

At the end of the experiments, the pups assigned to hyperoxia showed a shorter colon than those in the normoxic groups (Figure 2), indicating that the high oxygen level during early post-natal life alters colon development. Treatment with BRL37344 at 3 mg/kg significantly ameliorated—but did not completely abrogate—this hyperoxia-induced colon length reduction, whereas the lowest 1 mg/kg dose was ineffective. On the other hand, no differences in colon length were found in the normoxia-exposed rats, whether treated or not with BRL37344.

**Figure 3:**
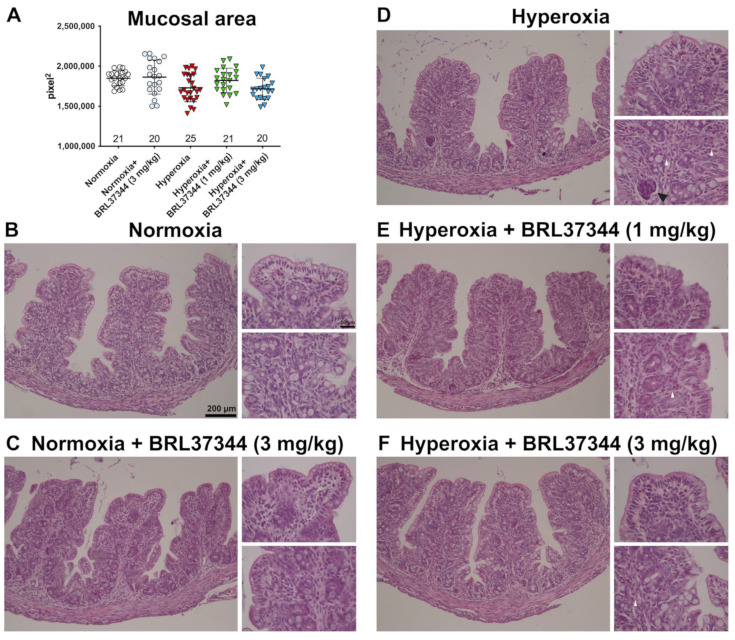
Histological evaluations of the effects of hyperoxia on proximal colon morphology. (**A**) Morphometrical analysis of mucosal area. (**B**–**F**) Representative images of H/E-stained sections. The general morphology of the proximal colon was almost preserved in all the animals of the different experimental groups, apart from scattered infiltrating mixed leukocytes in the pups exposed to hyperoxia (white arrowhead). A group of hyperchromatic, putatively apoptotic cells is evidenced by the black arrowhead in a colonic gland ending from a control hyperoxia-exposed pup. Values are expressed as mean ± S.D. Scale bars = 200 µm and 50 µm.

The effect of hyperoxia exposure on proximal colon morphology was also evaluated using morphometrical analysis. Mucosal area quantification performed on H/E-stained sections revealed no statistically significant differences between the experimental groups (Figure 3, panel A). Qualitative evaluations of the proximal colonic mucosa showed typically folded villi and an almost continuous lining epithelium in the rats from all the experimental groups (Figure 3, panels B–F). Minor signs of damage, namely hyperchromatic, putatively apoptotic cells in scattered colonic gland endings, were only detected in the hyperoxia-exposed control rats (Figure 3, panel D) [24]. As shown in Figure 3 (panels D–F), infiltrating mixed leukocytes were present in the colonic mucosa of all the hyperoxia-exposed rats, regardless of the treatment with BRL37344.

**Figure 4:**
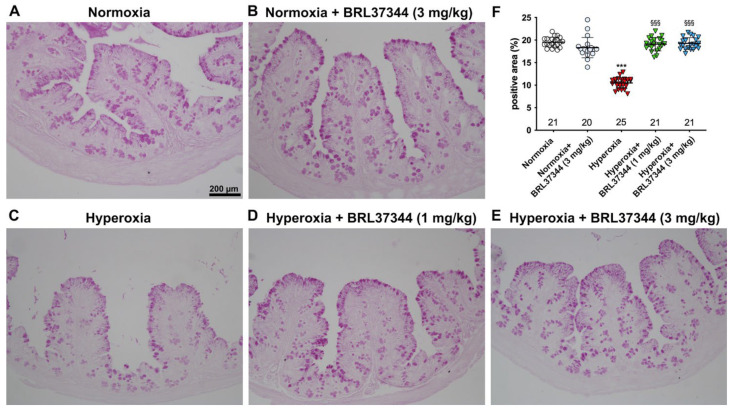
Histochemical evaluations of the effects of hyperoxia on the relative area of PAS^+^ mucin-producing goblet cells in the proximal colon. (**A**–**E**) Representative histologic images. (**F**) Densitometric analysis of PAS^+^ relative area, showing significantly reduced values upon exposure to hyperoxia (**A**,**C**,**F**), an effect prevented by administration of the β3-AR agonist BRL37344 at both doses (**A**,**D**–**F**). No alterations in the PAS^+^ relative area were detected upon the administration of BRL37344 to normoxia-exposed rats (**A**,**B**,**F**). Values are expressed as mean ± S.D. *** *p* < 0.001 hyperoxia versus normoxia; §§§ *p* < 0.001 hyperoxia + BRL37344 (1 mg/kg) or hyperoxia + BRL37344 (3 mg/kg) versus hyperoxia. Scale bar = 200 µm.

The effect of hyperoxia on mucin expression was then evaluated by a quantitative analysis of PAS-stained colon sections [25]. As reported in Figure 4, hyperoxia induced a statistically significant loss of the PAS^+^ relative area of goblet cells, suggesting reduced mucin production [24]. The administration of BRL37344 at both 1 mg/kg and 3 mg/kg to the hyperoxia-exposed pups was able to restore the PAS^+^ mucin tissue area to the levels of the normoxic pups (Figure 4, panels A, D, E, F). In contrast, its administration under normoxia did not influence the PAS^+^ mucin relative area (Figure 4, panels A, B, F).

### 3.4. Neuronal Chemical Coding in the Myenteric Plexus

**Figure 5:**
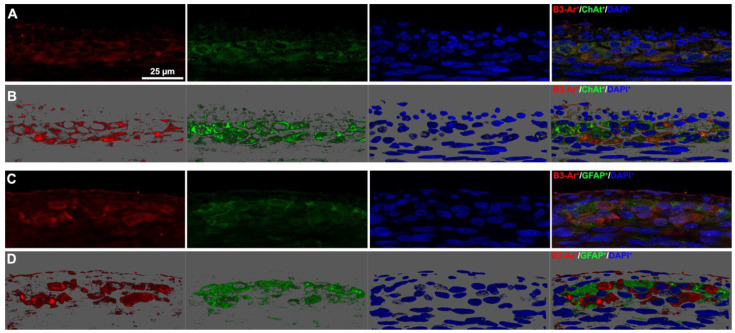
β3-AR-expressing cell types in the myenteric plexus. (**A**,**B**) Representative images of β3-AR-positive cells (red) and ChAT-positive neurons (green). (**B**) 3D deconvolution. (**C**,**D**) Representative images of β3-AR-positive cells (red) and GFAP-positive glial cells (green). (**D**) 3D deconvolution. Nuclei are counterstained blue with DAPI. Scale bar = 25 µm.

We then sought to identify the myenteric plexus cell types expressing β3-AR. As previously reported [20,26], the presence of this receptor was confirmed on ChAT^+^ cholinergic neurons (Figure 5, panels A and B), whereas no GFAP-positive glial cells were found to express β3-AR (Figure 5, panels C and D). This finding prompted us to focus on neuronal rather than glial alterations.

**Figure 6:**
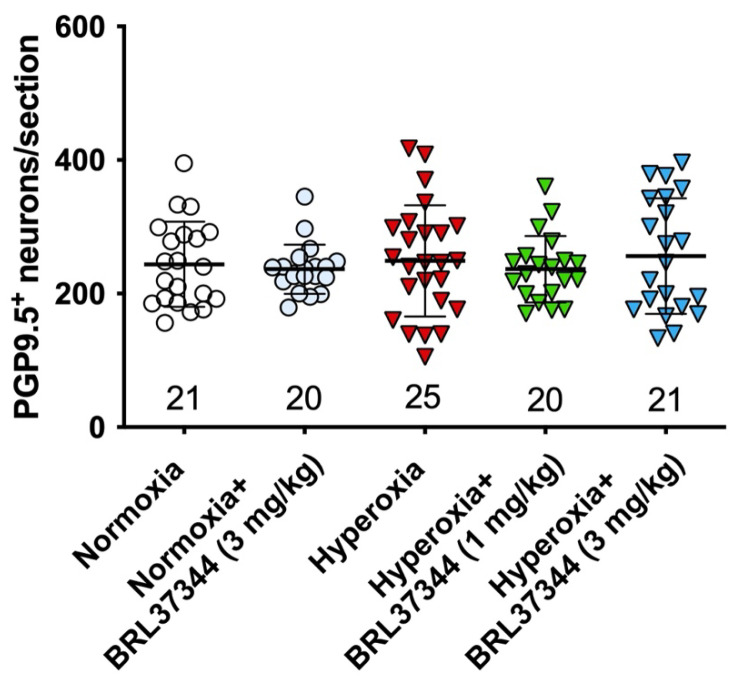
Quantification of total neurons in myenteric plexus. The pan-neuronal marker PGP 9.5 was used to identify and count the neurons in myenteric ganglia per section. No statistically significant differences were revealed between the experimental groups.

The impact of hyperoxia on the myenteric neuronal population in the proximal colon was first assessed by counting neuronal cell bodies expressing the pan-neuronal marker PGP9.5. No statistically significant differences were found in the total number of neurons between the experimental groups (Figure 6).

**Figure 7:**
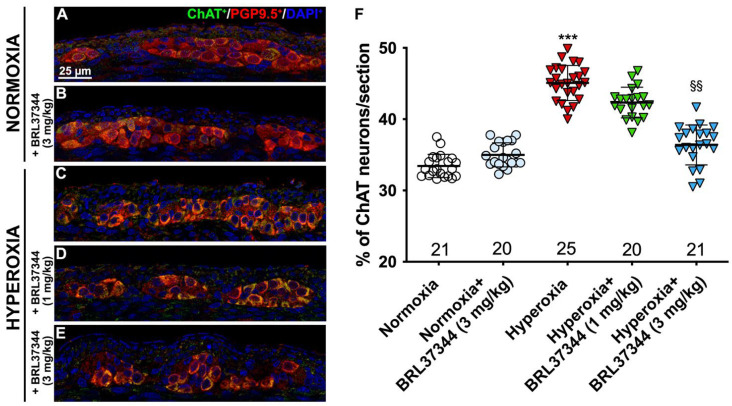
ChAT and PGP9.5 double immunolabelling in the myenteric ganglia. (**A**–**E**) Representative images of ChAT^+^ (green) and PGP9.5^+^ (red) neurons. Nuclei are counterstained blue with DAPI. (**F**) Analysis of ChAT/PGP9.5 percentage: compared with pups reared to normoxia, a statistically significant increase in ChAT^+^ neurons was detected in the control hyperoxia-exposed pups. Treatment with BRL37344 at 3 mg/kg completely prevented this alteration, whereas it was only partially effective at 1 mg/kg. No differences were found in the rats treated with the β3-AR agonist and untreated rats under normoxic conditions. Values are expressed as mean ± S.D. *** *p* < 0.001 hyperoxia versus normoxia; §§ *p* < 0.01 hyperoxia + BRL37344 (3 mg/kg) versus hyperoxia. Scale bar = 25 µm.

**Figure 8:**
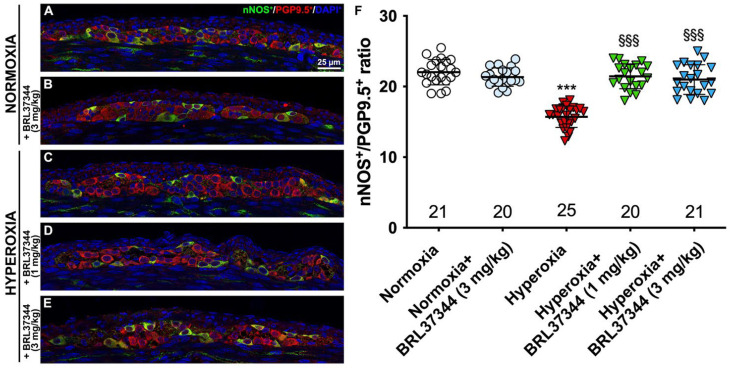
nNOS and PGP9.5 double immunolabelling in the myenteric ganglia. (**A**–**E**) Representative images of nNOS^+^ (green) and PGP9.5^+^ (red) neurons. Nuclei are counterstained blue with DAPI. (**F**) Analysis of nNOS/PGP9.5 percentage: compared with pups reared to normoxia, a statistically significant decrease in nNOS^+^ neurons was detected in the control hyperoxia-exposed pups. Treatment with BRL37344 at both doses prevented this alteration. No differences were found in the rats treated or not with the β3-AR agonist in normoxic conditions. Values are expressed as mean ± S.D. *** *p* < 0.001 hyperoxia versus normoxia; §§§ *p* < 0.001 hyperoxia + BRL37344 (1 mg/kg) or hyperoxia + BRL37344 (3 mg/kg) versus hyperoxia. Scale bar = 25 µm.

Although no differences were found in the total number of neurons in the myenteric plexus, hyperoxia did induce some alterations in neuronal chemical coding. ChAT- and nNOS-positive neurons were counted, and ChAT/PGP9.5 and nNOS/PGP9.5 percentages were calculated to further explore this aspect. Furthermore, SP expression in the myenteric ganglia was also measured. Compared with pups reared to normoxia, a statistically significant increase in ChAT^+^ neurons, accompanied by a robust reduction in nNOS^+^ neurons, was revealed in control hyperoxia-exposed pups (Figure 7 and Figure 8, Panels A, C, F). Treatment with BRL37344 at 3 mg/kg completely prevented these alterations, preserving the normal neuronal chemical coding, whereas the β3-AR agonist at 1 mg/kg dose was ineffective (Figure 7 and Figure 8, Panels A, C–F). No such changes were detected between the control and BRL37344-treated animals exposed to normoxia (Figure 7 and Figure 8, Panels A, B, F).

**Figure 9:**
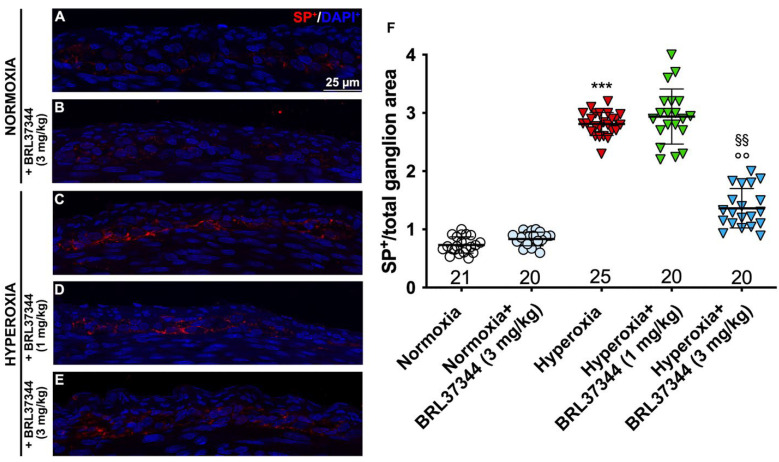
SP immunolabelling in myenteric ganglia. (**A**–**E**) Representative images of SP expression (red). Nuclei are counterstained blue with DAPI. (**F**) Morphometric analysis of SP-positive area per ganglion. Compared with normoxic control rats, those exposed to hyperoxia significantly increased SP^+^ nerve fibers. Treatment with BRL37344 at 3 mg/kg, but not at 1 mg/kg, exerted a partially protective effect. Values are expressed as mean ± S.D. *** *p* < 0.001 hyperoxia versus normoxia; §§ *p* < 0.01 hyperoxia + BRL37344 (3 mg/kg) versus hyperoxia; °° *p* < 0.01 hyperoxia + BRL37344 (3 mg/kg) versus normoxia. Scale bar = 25 µm.

We then evaluated SP expression to further investigate the oxygen-dependent modifications to the neuronal chemical coding of myenteric plexus. As shown in Figure 9 (Panels A, C, F), a statistically significant increase in the SP immunofluorescent area corresponding to nerve fibers was demonstrated in the rats from the hyperoxia control group compared with the normoxic ones. Treatment with BRL37344 at 3 mg/kg exerted a protective effect, even if it was unable to completely restore SP expression at the level of the normoxic controls (Figure 9, Panels A, C, E, F). Conversely, BRL37344 was ineffective at 1 mg/kg (Figure 9, Panels A, C, D, F). Once again, no differences were found in SP immunoreactivity in the rats treated with the β3-AR agonist under normoxic conditions and untreated rats (Figure 9, Panels A, B, F).

## 4. Discussion

Although it is well-known that neonatal exposure to hyperoxia alters the final stage of the maturation process of some organs, including lungs and eyes, ultimately leading to diseases such as BPD and ROP [8], minimal research has been performed to elucidate its role in post-natal gut intestinal development [5,8,9,27]. Furthermore, the available data mainly concern the hyperoxia-induced alterations in the small bowel, leaving those in the large intestine in shade. To correct this lack of knowledge, the present in vivo study first shows the alterations induced by high oxygen levels on the proximal colon in a well-characterized neonatal rat model [5,27] and, in addition, the beneficial effect of the moderately selective β3-AR agonist BRL37344. This compound is the most widely used among the β3-AR agonists, being 20-fold more selective for β3-AR than β2-AR [28] in human and displaying even a higher affinity for rodent β3-AR receptors [29]. However, more recently, it was also shown to act via β2-AR in some tissues, such as skeletal muscle [30]. Despite its moderate selectivity for the β3-AR, this compound was chosen for our study because it can be administered subcutaneously [31], whereas other, more selective, β3-AR agonists, such as mirabegron, are preferentially given intravenously or orally. As the animal model used for this study involves newborn rats, it does not allow for repeated intravenous or oral treatments. Taking into account the moderate selectivity of BRL37344 for the β3-AR, it is not possible to rule out that some of the preventive effects reported here may be also exerted via β2-AR.

The first remarkable finding of our study is that hyperoxia significantly reduced the number of resident cells expressing β3-AR in the proximal colon of rats. Although this result deserves further investigations and is insufficient to establish a direct correlation between hyperoxia and reduced β3-AR expression, it may contribute to a better understanding of the oxygen-dependent regulatory mechanism of β3-AR, which has been only investigated in low-oxygen conditions until now. It is acknowledged that this receptor is up-regulated by the hypoxic microenvironment, as occurs in the developing embryo during intra-uterine life [15,16,17] or during the hypoxic phase of retinopathy [32], but, to the best of our knowledge, little is known about the effect of hyperoxia on β3-AR expression, with the only relevant information being our recent report showing its downregulation on the murine ductus arteriosus after delivery when newborns are exposed to a more oxygenated environment as compared with that occurring in utero [18]. Although not conclusive, this report and the present finding that hyperoxia reduced the number of colonic cells strongly expressing β3-AR suggest that β3-ARs can be downregulated by high oxygen levels and may be involved in the developmental processes of various organs, including the colon, in close connection with ambient oxygen levels. In particular, when upregolated by hypoxia [12,15,16,17], β3-AR may stimulate the intra-uterine phases of morphogenesis. In contrast, its post-natal downregulation induced by exposure to normal blood oxygen levels upon ambient air breathing shuts the hypoxic phase down. At the same time, it promotes the final maturation process.

In this scenario, the second major finding of the present study, demonstrating that hyperoxia shortened colonic length while the administration of the β3-AR agonist BRL37344 (3 mg/kg) significantly prevented this reduction, becomes even more important. Since rodents have a brief gestation period and their relatively immature gut continues to develop for the first 14 days after birth [33], it is conceivable that abnormally elevated oxygen levels may alter the final stage of colonic development via changes in β3-AR expression. Of note, despite its beneficial effect in preventing colonic-length shortening, β3-AR agonist was unable to counteract the hyperoxia-induced body-weight loss. This effect is likely due to the impaired general health status of puppies induced by high levels of reactive oxygen species and related inflammation rather than to the well-known anorectic effects of β3-AR activation [34,35]. This hypothesis is also strengthened by the finding that the administration of BRL37344 in normoxia did not alter the body weight of the pups.

Another major finding of this study is that hyperoxia impairs the production of mucins by goblet cells within the lining epithelium of the proximal colon. This is accompanied by only minor alterations in the general histomorphology of this organ, at variance with the previous studies in a similar animal model showing that hyperoxia induces injury in the distal small intestine, mainly by disrupting the intestinal epithelial barrier [5,8]. This apparent discrepancy could be explained by considering that the gut can be regarded as a multi-task organ, as it is composed of regions with different structural characteristics to fulfill specific functions, which may react differently to the same stimulus [8]. Although histological analysis has shown a substantially normal surface epithelium, we know that further studies are required to ascertain whether hyperoxia may impair the barrier function of the proximal colon. The decrease in mucin production indicated by the observed reduction in PAS^+^-relative area is a marker of intestinal damage [24,36] and could be at least partially explained by a loss of goblet cells [24]. This finding is at variance with previous studies, which have reported an overproduction of mucins in rodent models of colonic injury as a reaction to dysmotility [25,37]. However, these animal models were designed to mimic intestinal disorders characterized by chronic inflammation and long-lasting treatments, whereas ours exploits a different noxious stimulus applied for a short time to immature animals. Such major differences may contribute to explaining the different findings regarding mucin production. Moreover, it is also known that in ulcerative colitis and Crohn’s disease, the two main clinical manifestations of inflammatory bowel disease, the alterations in mucus show opposite trends [38].

The third major finding of the present study is that hyperoxia has an in-depth influence on the post-natal development of the colonic myenteric plexus, while the administration of BRL37344 at 3 mg/kg significantly reduced this alteration, although not completely. The early post-natal period is characterized by the differentiation of the colonic myenteric neurochemical phenotype, which has a major impact on neuro-muscular function [39,40]. Despite some discrepancies concerning the individual contribution of different neuronal subpopulations [39,40,41], it is acknowledged that, in the first weeks after birth, cholinergic excitatory motoneurons, often colocalized with SP, and nitrergic motoneurons or interneurons, undergo relevant changes in their maturation [40]. Our study first shows that neonatal hyperoxia significantly alters neuronal chemical coding in the myenteric plexus, increasing both ChAT^+^ neurons and the SP immunoreactivity of nerve fibers while reducing the nNOS^+^ neurons without changing the number of neurons per ganglion. This observation agrees with our previous results from a model of diabetes-induced intestinal injury [25]. In contrast, other models of colonic disorders have shown neuronal loss or different alterations in neuronal subpopulations [36,42,43]. Our key finding that hyperoxia did not reduce the total number of neurons strongly suggests that β3-AR activation exerts a neuroprotective effect, not substantially preventing cell loss, as previously demonstrated in a model of retinal damage [44,45], but rather by improving the maturation process. Conceivably, the β3-AR agonist BRL37344 kept the neurochemical coding of the myenteric plexus almost unchanged by uncoupling its developmental process from the adverse effect of hyperoxia. Again, considering the moderate selectivity of BRL37344 for the β3-AR, it cannot be ruled out that the observed neuroprotective effect may be partially exerted by treatment via β2-AR agonism. Although this receptor has not been localized on the neuronal population of the myenteric plexus of the rat [46], it is also possible that BRL37344, by acting on neurons of the submucosal plexus, which instead express β2-AR, may have induced an indirect protective action through neurons. Furthermore, our findings support the previous observations that β3-AR is expressed by cholinergic neurons in the myenteric plexus [20,47]. At the same time, it provides the first evidence that this receptor is not expressed by GFAP-positive glial cells in the same ganglia. Although it is well-known that GFAP can label most, but not all, glial cells [48], our results do indicate that the preferential target of BRL37344 appears to be the neuronal rather than the glial gut ganglionic cell population. However, further studies are required to clarify this point, as well as to investigate the possible effects of its pharmacological targeting.

A final point deserving comment concerns the massive neonatal death induced by the administration of BRL37344 at the highest dose (6 mg/kg). The first possible explanation is that BRL37344 at 6 mg/kg is toxic. However, since it is well-known that β3-AR agonism activates lipolysis and causes hypophagia, it is equally possible that the combination of hyperoxia-induced body weight loss on the one hand, and abnormal feeding behavior and sub-lethal toxic effects of 6 mg/kg BRL37344 on the other hand, have resulted in a synergistic effect that is lethal to the newborns rats. Whichever the causes, for ethical reasons, we decided not to test this high dose of BRL37344 on the normoxic animals. To our knowledge, no literature is available in this regard. However, BRL37344 administered at a dose effective in preventing hyperoxia-induced alterations (3 mg/kg), did not show anorectic or toxic effects, nor did it modify any of the analyzed parameters in the normoxic rats. These results demonstrate that this compound has an effective therapeutic window at low to moderate doses.

There are some limitations to this study. First, as previously reported, given the exploratory nature of this research, the present findings should be further confirmed by a formal hypothesis-testing study. Additionally, the use of BRL37344 instead of other compounds with a higher selectivity for β3-AR does not allow for us to exclude the possible involvement of β2-AR in the protective effect against hyperoxia-driven colonic damage or in the post-natal maturation process of this organ. To address this issue, further experimental studies evaluating the effects of second-generation agonists such as mirabegron or using β3-AR knock-out animals are needed. Third, we did not take into account some relevant factors that may influence the colonic maturation processes, including microbial colonization, that would require different in vivo approaches. Finally, due to interspecies differences, the reported prominent impact of hyperoxia on the gut and the effects of BRL37344 were purposely verified in a rather extreme experimental condition, which does not occur in preterm newborns, and no data are available with lower oxygen tension. To promote the translation from bench to bedside, a further investigation using animal models more representative of the clinical settings is needed.

Taking into account these limitations and the fact that extrapolation of the results from studies on animal models to humans must be treated cautiously, the current findings may still potentially have clinical implications, especially for premature infants. Preterm newborns are at high risk of developing diseases such as bronchopulmonary dysplasia, retinopathy of prematurity, periventricular leukomalacia and necrotizing enterocolitis, which, despite presenting with different clinical symptoms and signs, may share closely related pathogenic pathways. Indeed, premature exposure of immature organs to ambient air, yielding a relatively hyperoxic environment compared with physiologic intra-uterine hypoxia, could alter their developmental process, decreasing β3-AR expression and thus impairing its role in organ maturation. In this context, the availability of effective and safe β3-AR agonists in the therapeutic repertoire of neonatal medicine could represent a new resource for the prevention or treatment of these severe complications of prematurity. Concerning the intestine, the close relationship between hyperoxia and intestinal damage [49], and the consequent susceptibility to developing NEC, is well-known [27]. Therefore, the agonism of β3-AR could represent an ideal pharmacological target to protect intestinal development and prevent NEC.

## 5. Conclusions

In conclusion, our study shows that hyperoxia induces profound alterations in the post-natal development of the proximal colon, conceivably reducing β3-AR expression. It also offers the first evidence that the administration of moderately selective β3-AR agonist BRL37344 can significantly prevent such alterations. These findings further pave the way for the pharmacological targeting of β3-AR as a new therapeutic option for colonic diseases caused by hyperoxia-impaired development, typical disorders of the preterm infant syndrome. More generally, the β3-AR agonism could represent a new pharmacological approach to promote organ maturation in prematurity-related diseases (bronchopulmonary dysplasia, retinopathy of prematurity, necrotizing enterocolitis, periventricular leukomalacia). This exciting perspective deserves further investigation.

## 6. Patent

The University of Pisa and the University of Florence have filed a patent application on the effect of β3-AR agonism in promoting neuronal maturation and preserving neuronal chemical coding. L.F., P.N. and A.P. are listed as inventors on this patent.

## Figures and Tables

**Table 1:** Primary and secondary antisera used in immunofluorescence analysis.

Antigen	Species	Source	Catalog Number	RRID	Concentration
		Primary Antisera			
β3-AR	Rabbit	GeneTex (Irvine, CA, USA)	GTX70685	AB_375242	1:300
ChAT	Chicken	GeneTex (Irvine, CA, USA)	GTX85450	AB_10622340	1:200
GFAP	Chicken	Abcam (Cambridge, UK)	ab4674	AB_304558	1:1000
nNOS	Rabbit	GeneTex (Irvine, CA, USA)	GTX133403	AB_2886968	1:500
PGP 9.5	Mouse	GeneTex (Irvine, CA, USA)	GTX634797	AB_2888478	1:300
SP	Goat	Santa Cruz Biotech (Santa Cruz, CA, USA)	AB_661439	AB_661439	1:100
		Secondary Antisera			
Alexa Fluor 488	Donkey	Jackson ImmunoResearch (Ely, UK)	711-545-152	AB_2313584	1:175
Alexa Fluor 488	Donkey	Jackson ImmunoResearch (Ely, UK)	703-545-155	AB_2340375	1:175
Alexa Fluor 594	Donkey	Jackson ImmunoResearch (Ely, UK)	711-585-152	AB_2340621	1:175
Alexa Fluor 594	Bovine	Jackson ImmunoResearch (Ely, UK)	805-585-180	AB_2340884	1:175
Alexa Fluor 594	Sheep	Jackson Immunoresearch (Ely, UK)	515-585-062	AB_2340337	1:175

β3-AR = beta 3 adrenergic receptor; ChAT = choline acetyltransferase; GFAP = glial fibrillary acidic protein. nNOS = neuronal nitric oxide synthase; PGP 9.5 = proteine gene product 9.5; SP = Substance P.

**Table 2:** Body weight of rats at birth (P0) and at the end of the experimental period (P14).

Experimental Group	Pups/Group	Age (d)	Body Weight (g)	Age (d)	Body Weight (g)
Normoxia	21	0	6.5 ± 0.6	14	33.5 ± 1.7
Normoxia + BRL37344 (3 mg/kg)	20	0	6.5 ± 0.7	14	32.1 ± 1.9
Hyperoxia	25	0	6.4 ± 0.7	14	21.4 ± 1.7 ***
Hyperoxia + BRL37344 (1 mg/kg)	22	0	6.5 ± 0.9	14	21.8 ± 1.6 °°°
Hyperoxia + BRL37344 (3 mg/kg)	21	0	6.6 ± 0.9	14	23.5 ± 1.7 °°°

Values are expressed as mean ± S.D. *** *p* < 0.001 hyperoxia versus normoxia. °°° *p* < 0.001 hyperoxia + BRL37344 (1 mg/kg) or hyperoxia + BRL37344 (3 mg/kg) versus normoxia.

## Data Availability

The datasets generated during and/or analyzed during the current study are available from the corresponding author upon reasonable request.

## Supplementary Materials

The following supporting information can be downloaded at: https://www.mdpi.com/article/10.3390/biom13121755/s1, Figure S1: Validation of the anti-β3-AR antibody for flow cytometric analysis. Flow cytometric analysis showed no staining for the β3 receptor in the epidermis as the signal appears exactly like the unlabeled sample. On the contrary, compared to the epidermal sample, the brown adipose tissue showed a high positivity demonstrated by a strong shift in fluorescent signal.; Figure S2: Validation of the anti-β3-AR antibody for immunofluorescent analysis. β3-AR immunolabelling in the rat epidermal (top) and fat (bottom) tissue samples taken as negative and positive controls, respectively, of the β3-AR antibody selected for staining the colon samples in Figure 5.
