# Supplementary material for: β3 Adrenoceptor Agonism Prevents Hyperoxia-Induced Colonic Alterations
Source: Biomolecules. 2023 Dec 6;13(12):1755. doi: 10.3390/biom13121755 (PMC10741994; doi:10.3390/biom13121755)
Supplement: Supplementary file 1 [file biomolecules-13-01755-s001.zip › biomolecules-2667139-supplementary.pdf]

Supplementary Figure legends

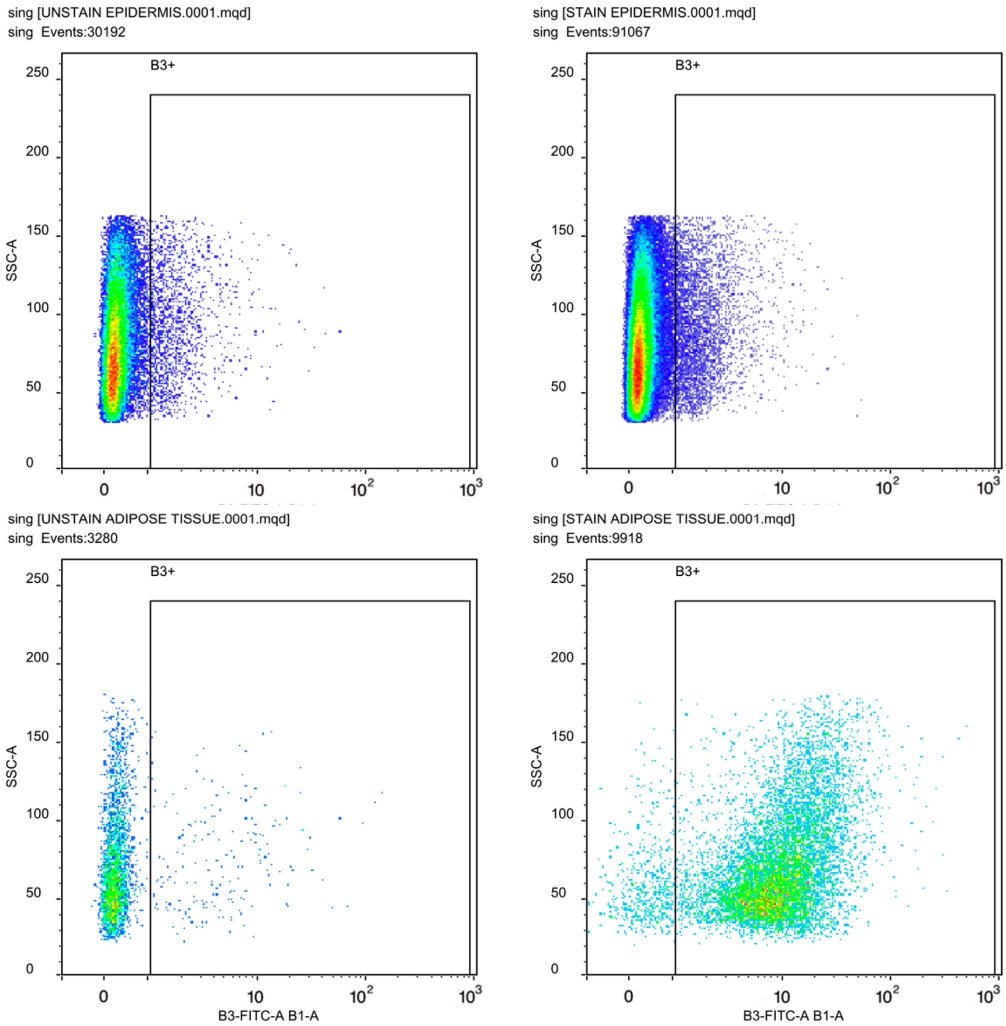

Figure S1. Flow cytometric analysis showed no staining for the β3 receptor in the epidermis as the signal appears exactly like the unlabeled sample. On the contrary, compared to the epidermal sample, the brown adipose tissue showed a high positivity demonstrated by a strong shift in fluorescent signal**.**

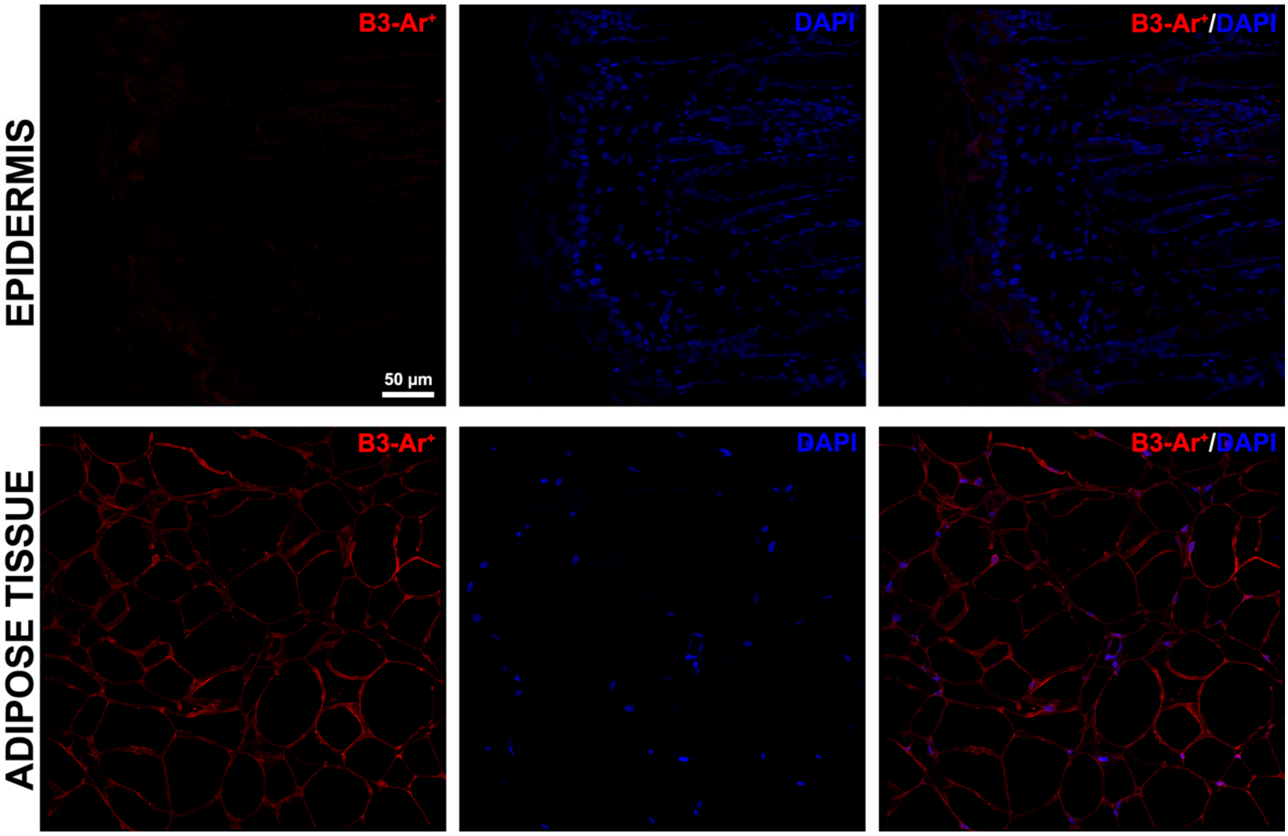

Figure S2. β3-AR immunolabelling in the rat epidermal (top) and fat (bottom) tissue samples taken as negative (doi: 10.1038/s41388-019-0993-1) and positive controls (doi: 10.3390/cells8040357), respectively, of the β3-AR antibody selected for staining the colon samples in Figure 5.
